# Pet and turtle: DNA barcoding identified twelve Geoemydid species in northeast India

**DOI:** 10.1080/23802359.2018.1467215

**Published:** 2018-04-26

**Authors:** Shantanu Kundu, Vikas Kumar, Boni Amin Laskar, Kaomud Tyagi, Kailash Chandra

**Affiliations:** a Centre for DNA Taxonomy, Molecular Systematics Division, Zoological Survey of India, Kolkata, India;; b Freshwater Biology Regional Centre (FBRC), Zoological Survey of India, Hyderabad, India

**Keywords:** Pet-trade, threatened taxa, DNA sources, mitochondrial DNA, conservation genetics

## Abstract

Geoemydid turtles are one of the most imperilled fauna on the planet, with nearly half of them are threatened with extinction due to bushmeat crisis, traditional medicine, and the illegal pet trade. Classical taxonomy often fails to identify the pet-kept turtle specimens due to amorphous form, unusual shell colouration owing to poor storage in captivity or intensely tinted for high demanding value. The DNA barcoding technique has evidenced as a supportive tool for accurate species identification in systematics research and discerned the nameless taxa in forensic sciences. We tested the effectiveness of DNA barcoding tools for identifying the pet-kept Geoemydid turtle in northeast India. The 36 generated sequences are readily delineated into 12 Geoemydid species using molecular data. The overall mean genetic distance of the studied Geoemydid turtles dataset is 15.3% and ranges from 3.4% to 22.6% between the species. The NJ, ML and Bayesian phylogeny also resulted monophyletic clustering and discriminated all the studied species. The present study contributes DNA barcode sequences of Geoemydid turtles in the global database and also affirms the on-going illegal pet trade of highly threatened species in northeast India.

## Introduction

Geoemydid turtles are one of the most ornamental and highly threatened Chelonian groups in the world (van Dijk et al. 2000; Buhlmann et al. 2009). Since the sixteenth century, the animals have been associated with the human for several mythological beliefs and recreational purposes (Klemens 2000; Fordham et al. 2007; Chen et al. 2009). The family Geoemydidae comprises of 71 species within 19 genera and two subfamilies worldwide (Turtle Taxonomy Working Group 2017). Among them, 13 species within eight genera are reportedly distributed throughout northeast India (Das 2001; Buhlmann et al. 2009). They mostly inhabit in the wild vegetation and have the burrowing nature of living under the leaf litters with exception of safe exposure to sunlight for basking. Most of them are dwellers surrounding freshwater ecosystems, and a few are adapting to estuarine or terrestrial habitats (Ernst et al. 2000). The genera *Batagur* and *Hardella* are reported to be confined to the river, while *Pangshura*, *Melanochelys*, *Geoclemys*, *Cuora*, *Cyclemys*, and *Morenia* are found in small hill streams or stagnant water bodies (Praschag et al. 2007).

Northeast India is also known as a turtle trading hub and dozens of turtles are being hunted by the local peoples in every year. The peoples in this region consume turtle meat for therapeutic practices, use burning ash of the turtle shell as traditional medicine, keep the dry shell as holy things and live specimens as pets. Thus, the overexploitation of turtles has been causing massive declination of the wild population in this region (Kundu et al. 2013, 2015, 2016, 2018). Several baseline studies have been fulfilled to know the morphology, genetic information, and distribution pattern of wild living Geoemydid turtles in northeast India (Das 2001; Praschag et al. 2007; Kundu et al. 2016). However, investigation of pet-kept turtles and their genetic identity was never being assessed in this region to estimate illegitimate threats on this threatened taxa.

Besides, the practice with classical taxonomy sometimes fails to identify the pet-kept turtle species due to the absence of important phenotypic characters. At this juncture, the interventions of molecular approaches are worthwhile to identify the species (Alacs et al. 2007). The molecular tools, DNA barcoding is evidenced as a supporting method in classical taxonomy and systematics research and have rendered clearer understanding to identify the extant fauna throughout the globe (Hebert et al. 2003). The DNA based species identification has been attempted to recognize the freshwater turtles worldwide, including India (Reid et al. 2011; Kundu et al. 2013, 2015, 2016, 2018). Nevertheless, the DNA barcode information of Indian Geoemydid turtles is still deficient in the global database. Thus, the study aimed to identify the pet-kept Geoemydid turtles through DNA barcoding. The generated DNA data would enrich the global database as well as helps to identify Geoemydid species and track the illegal turtle trade hereafter.

## Materials and methods

### Study site, ethical concern, and sampling

**Figure 1.**
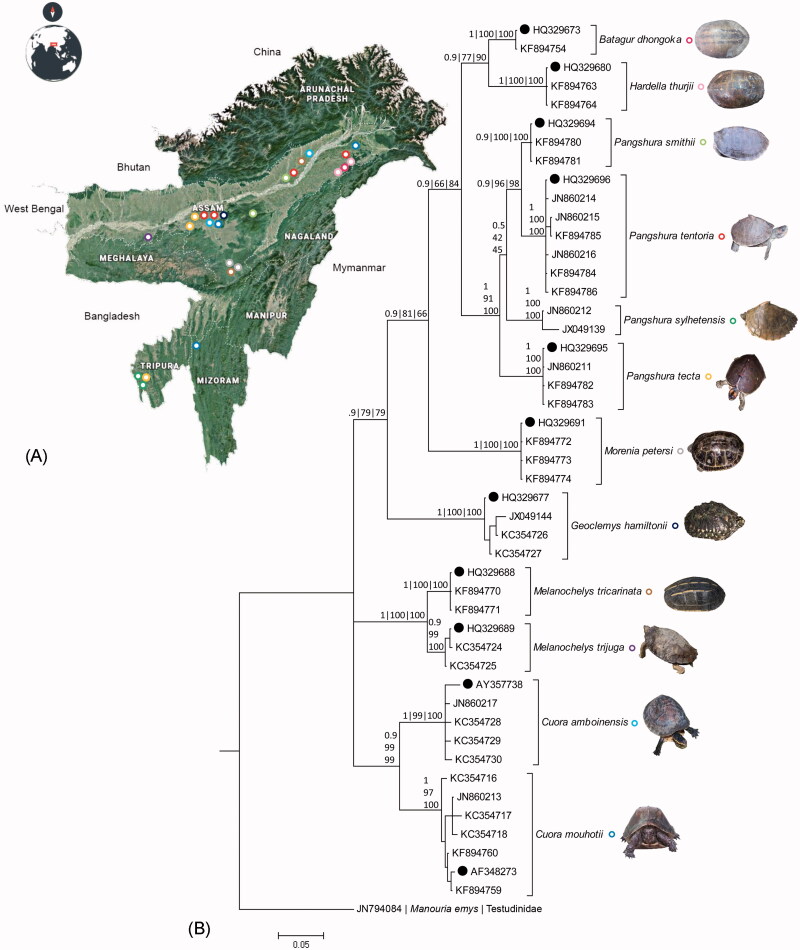
(A) The topographic map showing the collection localities of studied pet-kept Geoemydid turtles in northeast India. The original template of the map and world map inset used here is copied from Google Maps (https://www.google.co.in/maps). The map was edited manually in Adobe Photoshop CS 8.0. (B) Bayesian phylogeny based on the partial mtCOI gene of the studied Geoemydid turtles. Bayesian posterior probabilities and bootstrap values of both ML and NJ are superimposed horizontally and vertically with the nodes. Sequences marked by dots represent the published reference sequences acquired from GenBank database. The different colour circles embedded beside the species name in the phylogeny represents the collection localities of samples shows in the topographic map of northeast India. Representative species photographs were superimposed with the respective species clades in the phylogeny.

The pet-kept Geoemydid turtles were surveyed throughout northeast India after acquiring prior permission from the wildlife authority during 2010–2013. Most of the studied samples were collected from the villages of Assam state and a few individuals were collected from Tripura and Mizoram (Table 1, Figure 1A). The animals were handled with sufficient care and a meagre amount of blood or saliva was collected from each animal; solely for scientific research. The dry tissue samples were also collected from the carapace shells, are being kept after consuming the meat in the rural houses. The blood samples were collected from the hind limb by insulin syringe and stored into EDTA containing vial. The saliva samples were collected from the buccal cavity by swabbing and dipped into the 200 µl TES buffer (50mM Tris-HCl, 25mM EDTA, 150 mM NaCl). The samples were stored at 4 °C in the field and subsequently at −20 °C in the laboratory before DNA based investigation.

**Table 1.** Collection information of the Geoemydid turtles kept as pet in northeast India and BLASTn results of the generated mtCOI sequences depicting species identity.

Sl. No.	Voucher ID	Accession No.	BLASTn result	Species identification	Lat lon	Locality	DNA Source
1	AUTK81	KF894786	98%–100% with *Kachuga tentoria* (HQ329696)	*Pangshura tentoria*	26.39 N 92.47 E	Bechamari, Assam	Dry tissue
2	AUTK72	KF894785		*Pangshura tentoria*	26.39 N 92.47 E	Bechamari, Assam	Blood
3	AUTK71	KF894784		*Pangshura tentoria*	26.98 N 94.37 E	Chinatoli Chapori, Assam	Dry tissue
4	AUTK99	JN860216		*Pangshura tentoria*	26.38 N 92.48 E	Rou-Mari Bill, Assam	Saliva
5	AUTK80	JN860215		*Pangshura tentoria*	26.38 N 92.48 E	Rou-Mari Bill, Assam	Blood
6	AUTK21	JN860214		*Pangshura tentoria*	27.30 N 95.22 E	Merbill, Assam	Dry tissue
7	AUTK45	KF894783	98%–100% with *Kachuga tecta* (HQ329695)	*Pangshura tecta*	26.21 N 92.41 E	Rupaibari, Assam	Saliva
8	AUTK44	KF894782		*Pangshura tecta*	26.39 N 92.47 E	Bechamari, Assam	Blood
9	AUTK17	JN860211		*Pangshura tecta*	23.50 N 91.25 E	Nabadwipchandranagar, Tripura	Saliva
10	AUTK47	KF894781	99% with *Kachuga smithii* (HQ329694)	*Pangshura smithii*	26.98 N 94.37 E	Chinatoli Chapori, Assam	Saliva
11	AUTK46	KF894780		*Pangshura smithii*	26.35 N 93.44 E	Borpung, Assam	Saliva
12	SGSKD-T104	JX049139	93% with *Kachuga smithii* (HQ329694)	*Pangshura sylhetensis*	23.50 N 91.25 E	Nabadwipchandranagar, Tripura	Dry tissue
13	AUTK103	JN860212		*Pangshura sylhetensis*	23.50 N 91.25 E	Nabadwipchandranagar, Tripura	Saliva
14	AUTK105	KC354730	98%–100% with *Cuora amboinensis* (AY357738)	*Cuora amboinensis*	27.29 N 94.54 E	Khalihamari, Assam	Saliva
15	AUTK102	KC354729		*Cuora amboinensis*	27.29 N 94.54 E	Khalihamari, Assam	Blood
16	AUTK100	KC354728		*Cuora amboinensis*	26.38 N 92.48 E	Rou-Mari Bill, Assam	Dry tissue
17	AUTK101	JN860217		*Cuora amboinensis*	27.29 N 94.54 E	Khalihamari, Assam	Saliva
18	AUTK33	KC354718	98%–100% with *Pyxidea mouhotii* (AF348273)	*Cuora mouhotii*	26.38 N 92.48 E	Rou-Mari Bill, Assam	Dry tissue
19	AUTK32	KC354717		*Cuora mouhotii*	26.38 N 92.48 E	Rou-Mari Bill, Assam	Saliva
20	AUTK22	KC354716		*Cuora mouhotii*	27.30 N 95.22 E	Merbill, Assam	Dry tissue
21	AUTK66	KF894760		*Cuora mouhotii*	24.13 N 92.40 E	Zamuang, Mizoram	Saliva
22	AUTK16	KF894759		*Cuora mouhotii*	24.13 N 92.40 E	Zamuang, Mizoram	Dry tissue
23	AUTK23	JN860213		*Cuora mouhotii*	27.30 N 95.22 E	Merbill, Assam	Dry tissue
24	AUTK95	KC354727	98%–100% with *Geoclemys hamiltonii* (HQ329677)	*Geoclemys hamiltonii*	26.38 N 92.48 E	Rou-Mari Bill, Assam	Saliva
25	AUTK94	KC354726		*Geoclemys hamiltonii*	26.38 N 92.48 E	Rou-Mari Bill, Assam	Saliva
26	SGSKD-T97	JX049144		*Geoclemys hamiltonii*	26.38 N 92.48 E	Rou-Mari Bill, Assam	Dry tissue
27	AUTK84	KC354725	99%–100% with *Melanochelys trijuga* (HQ329689)	*Melanochelys trijuga*	26.11 N 91.44 E	Jalnamdani, Assam	Saliva
28	AUTK83	KC354724		*Melanochelys trijuga*	26.11 N 91.44 E	Jalnamdani, Assam	Saliva
29	AUTK37	KF894771	99%–100% with *Melanochelys tricarinata* (HQ329688)	*Melanochelys tricarinata*	25.10 N 93.00 E	Doiheng, Assam	Dry tissue
30	AUTK36	KF894770		*Melanochelys tricarinata*	27.28 N 94.54 E	Khalihamari, Assam	Blood
31	AUTK68	KF894754	98% with *Batagur dhongoka* (HQ329673)	*Batagur dhongoka*	27.20 N 95.32 E	Namrup, Assam	Dry tissue
32	AUTK42	KF894774	99%–100% with *Morenia petersi* (HQ329691)	*Morenia petersi*	25.15 N 93.08 E	Mahur, Assam	Dry tissue
33	AUTK41	KF894773		*Morenia petersi*	25.10 N 93.00 E	Doiheng, Assam	Saliva
34	AUTK40	KF894772		*Morenia petersi*	25.15 N 93.08 E	Mahur, Assam	Saliva
35	AUTK50	KF894764	98%–100% with *Hardella thurjii* (HQ329680)	*Hardella thurjii*	27.17 N 95.40 E	Namrup, Assam	Dry tissue
36	AUTK48	KF894763		*Hardella thurjii*	27.17 N 95.40 E	Namrup, Assam	Dry tissue

The voucher samples were stored at Assam University, Silchar, Assam.

### DNA isolation, PCR, and sequencing

The total genomic DNA was extracted followed by QIAamp DNA Mini Kit standard protocol. The published primer pair, FishF1-5′TCAACCAACCACAAAGACATTGGCAC3′ and FishR1-5′TAGACTTCTGGGTGGCCAAAGAATCA3′ (Ward et al. 2005) was used for amplification of partial mitochondrial cytochrome c oxidase subunit I (mtCOI) (∼650 bp) gene segment in a Veriti^®^ Thermal Cycler (Applied Bio systems, Foster City, CA). The 25 µl PCR mixture contains 10 pmol of each primer, 100 ng of DNA template, 1 × PCR buffer, 1.0–1.5 mM of MgCl2, 0.25 mM of each dNTPs, and 0.25 U of Platinum Taq DNA Polymerase High fidelity (Invitrogen, Life Science Technologies). PCR conditions were: initial denaturation at 94 °C (2 min) followed by 30 cycles at 94 °C (45 s), 50 °C (45 s), and 72 °C (1 min), and a final elongation at 72 °C (8 min). The PCR amplified products were checked in 1% agarose gel containing ethidium bromide (10 mg/ml). Further, the PCR products were purified using QIAquickR Gel extraction kit (QIAGEN Inc., Germantown, MD), and cycle sequencing products were cleaned by using standard BigDye X Terminator Purification Kit (Applied Biosystems, Foster City, CA). Sequencing was done bi-directionally in 48 capillary array 3730 DNA Analyzer (Applied Biosystems, Foster City, CA) following Sanger sequencing methods.

### DNA barcode sequence quality control measures and analysis

The generated chromatograms that represent sequences of both DNA strands were obtained for each sample. The noisy sequences were trimmed at both end and greater than 2% ambiguous bases were discarded from the generated chromatograms, using a quality value of >40 for bidirectional reads. The SeqScape software version 2.7 (Applied Biosystems Inc., Foster City, California, USA) was used to analyze to obtain the consensus sequences from the forward and reverse chromatograms. The sequences were submitted to the GenBank database for acquiring accession numbers. The homology search of the generated sequences was performed through nucleotide BLASTn search in the GenBank database. Based on the similarity search, the reference sequences showing highest identity matches for each of the studied species (*n* = 11) were also retrieved from the GenBank. The generated and acquired sequences were aligned with the ClustalX program (Thompson et al. 1997) to make a comprehensive dataset with equal length and common start position. The mean genetic divergences were calculated using Kimura 2 parameter (K2P) and the best evolutionary model (HKY + G) was selected with the lowest Bayesian information criterion (BIC) score (7490.52) by MEGA6.0 (Tamura et al. 2013). The DAMBE5 software was used to test the sequence substitution saturation of mtCOI gene within the studied Geoemydid species (Xia 2013). Phylogenetic analysis was performed under the optimality criteria of Neighbour-Joining (NJ) and Maximum Likelihood (ML) by using PAUP* 4.0b10 (Swofford 2002) with 1000 bootstrap support and Bayesian analysis (BA) using MrBayes 3.1 (Ronquist and Huelsenbeck 2003). For BA, Markov Chain Monte Carlo (MCMC) was performed with four chains for 1,000,000 generations, with trees sampled every 100 generations (the first 1000 trees were discarded as ‘burn in’). MCMC analysis was stationary when maximum standard deviation of split frequencies reached below 0.01 and potential scale reduction factor (PSRF) approached 1.0. Sequence of *Manouria emys* (family: Testudinidae) was incorporated to the dataset as the out-group in the Phylogenetic analysis.

## Results and discussion

The molecular taxonomy successfully delimitated most of the globally distributed extant Geoemydid species (Spinks et al. 2004; Praschag et al. 2007). The utility of mtCOI gene also successfully tested to identify the Geoemydid species (Reid et al. 2011; Kundu et al. 2016). Therefore, having found the competency of this molecular tool in accurate species identification and delineation, it is essential to generate DNA barcode data (Ihlow et al. 2016). The pet turtles are generally kept alive inside the small artificial water tank, barrel or aquarium without proper management and the shells are often intensely tinted by colours or chemicals for recreation purposes or high demanding commercial value. Thus, due to the lack of stable morphological characters, the classical taxonomy has its limitations to identify the pet-kept species occasionally. However, we have not observed any unusual morphological characters in the studied specimens which led to assume the possible hybridization and assure through more molecular markers. In this context, the current study dealt with the DNA data of pet-kept Geoemydid species collected from northeast India for accurate species-level identification and genetic distinctiveness. The study generated a total of 36 DNA barcode data of 12 pet-kept Geoemydid turtles collected from northeast India. The 11 samples shows 98%–100% identity match with the reference sequences in the GenBank databases and confirmed as *P. tentoria* (*n* = 6), *P. smithii* (*n* = 2), and *P. tecta* (*n* = 3). The two generated sequences shows 93% similarity with *P. smithii*, which infers an insignificant identity match due to lack of reference barcode in the database. In order to resolve this issue, the specimens were revisited and examined carefully to collect the necessary morphological data which finally led to identifying the species as *P. sylhetensis*. This study provides the first DNA barcode data information for *P. sylhetensis* from its known distribution region in northeast India. This contribution in the global database further assists to estimate the deep phylogenetic relationship among *Pangshura* congeners and intraspecific genetic distance from other geographical areas. The other 25 samples also shows 98%–100% similarity with the reference sequences in the GenBank database and identified as *Batagur dhongoka* (*n* = 1), *Hardella thurjii* (*n* = 2), *Morenia petersi* (*n* = 3), *Geoclemys hamiltonii* (*n* = 3), *Cuora amboinensis* (*n* = 4), *C. mouhotii* (*n* = 6), *Melanochelys tricarinata* (*n* = 2), and *M. trijuga* (*n* = 2). Thus, the present investigation recorded three endangered, five vulnerable, two lower risk/least concern, and two lower risk/near threatened Geoemydid turtles in northeast India (IUCN 2018). Due to the lack of knowledge about the species, their habitats, and proper awareness, the rural peoples in this region are often hunting the highly threatened freshwater turtles unknowingly during fishing practice or dry leaf and wood collection in wild. After interaction with the local peoples, the study also perceived that the population of lower risk categorized species in IUCN red list of threatened species are becoming rare in the wild. The overall mean genetic distance of the studied Geoemydid turtles dataset is 15.3% and ranges from 3.4% to 22.6% between the species. The genus *Pangshura* shows 3.4%–7.8% genetic distance between four known species in the dataset, whereas *Cuora* and *Melanochelys* with their two congeners show 8.3% and 4.2% genetic distance, respectively. In the total dataset, the six studied species, *P. smithii*, *P. tecta*, *B. dhongoka*, *H. thurjii*, *M. petersi*, and *M. tricarinata* shows 0% genetic divergence within the species in this current dataset. Further, the other six species, *P. tentoria*, *P. sylhetensis*, *G. hamiltoni*, *C. amboinensis*, *C. mouhotii*, and *M. trijuga* shows 0.2%, 2%, 1.2%, 2.1%, 1% and 0.4% within species genetic distance, respectively. The less genetic divergence (0%) within few studied species revealed their restrict gene pool in the studied locality, which warrants further sampling throughout their known distribution to perceive more clear understanding. The high genetic distance (≥2%) within the group of *P. sylhetensis* and *C. amboinensis* depicts the possible cryptic diversity within the species. The four congeners of *Pangshura*, *P. tentoria*, *P. smithii*, *P. sylhetensis*, and *P. tecta* shows 3.4%–7.8% genetic divergence between the species. Further, the *C. amboinensis* and *C. mouhotii* shows 8.3% and *M. tricarinata* and *M. trijuga* shows 4.2% genetic distance between the species in the current dataset. Sequence saturation analysis of mtCOI gene of the studied sequences showed the increase of frequency of both transitions and transversions linearly along with the divergence value. The NJ, ML and Bayesian phylogeny of the studied dataset shows cohesive monophyletic clustering of all the studied species with 100 bootstrap supports and 0.9–1 posterior probability (Figure 1B). The database reference sequences of the representative species also depicted cohesive clustering with the generated sequences in the phylogenetic tree.

The accurate identification of any taxa is vital before initiating any other allied assessment, i.e. physiology, ecology, behaviour, population estimation, and conservation. To assess the population structure of any species, researchers are not only concerned about the wild living individuals but also concerned about their threats estimation, records of enforcement seizures, pet trade etc. within and beyond their known geographical regions (Alacs et al. 2007; Mendiratta et al. 2017). For example, many new Geoemydid species have been described from the food and pet markets in the last two decades (Kou 1989; Parham and Shi 2001) and the genetic data corroborated the presence of three non-native turtles in northeast India kept as pets (Kundu et al. 2016). Thus, the present review of pet-kept Geoemydid turtles through molecular approaches has not only identified the species but also estimated illegitimate threats, pet trade scenario in the studied area and contributed genetic information in the global database. The results specified the serious concern in view of illegal wildlife hunting as well as protection of threatened Geoemydid turtle population in natural settings. The current approach can be useful for monitoring trade, the origin of seizures, and directing enforcement to protect overexploited turtle populations in the future.
